# Managing an open nasofrontal encephalocele after birth

**DOI:** 10.1007/s00381-022-05620-6

**Published:** 2022-11-10

**Authors:** Nicolas Apostolou, Daniel Gräfe, Matthias Knüpfer, Matthias Krause

**Affiliations:** 1 Department of Pediatric Neurosurgery, University Hospital Leipzig, Liebigstrasse 20, 04103 Leipzig, Germany; 2 Division of Neuroradiology, University Hospital Leipzig, Liebigstrasse 20, 04103 Leipzig, Germany; 3 Department of Neonatology, University Hospital Leipzig, Liebigstrasse 20, 04103 Leipzig, Germany

**Keywords:** Case report, Encephalocele, Nasofrontal

## Abstract

Encephaloceles are relatively uncommon in western countries. Most of the reported cases involve occipital encephaloceles. Open frontal encephaloceles comprise a rare entity. Most of them will be detected during early prenatal diagnostic, whereas the majority of the pregnancies will be terminated after the consent of the parents. Open frontal encephaloceles pose a great challenge to neurosurgeons as well as anesthesiologists, as these infants present with a microcephaly, non-physiological intracranial anatomy, and low birth weight, thus making the infant prone to excessive blood loss, hypothermia, and death. Neonates born with an incomplete cutaneous coverage are exposed to an imminent threat to life due to the risk of meningitis, necessitating surgical repair in the first days of life. We represent a rare case of an open nasofrontal encephalocele managed surgically in the first day of life. Surgery did not influence the neurological outcome of the patient.

## Introduction

Encephalocele is usually a congenital type of neural tube defect (NTD), where a sac containing brain/meninges/cerebrospinal fluid (CSF) forms outside the skull through a bone defect. On occasions, acquired, encephaloceles may result from trauma, tumors, or iatrogenic injury. If the sac is formed by protrusion of meninges and CSF, it is appropriately called a meningocele, but it is called an encephalocele when it contains brain tissue [1]. Encephaloceles are classified according to the anatomical defect location and the herniated contents [2, 3].

Surgical repair of the encephalocele does not require always an immediate surgical intervention. In the case of an incomplete cutaneous coverage, a rapid surgery is required due to the high risk of meningitis [1, 4–8]. Approximately 70 to 90% of encephaloceles involve the occipital area [9–11].

Frontoethmoidal encephaloceles are extremely rare, with an incidence of 1 in 35,000 live births [5]. They result from CNS herniation through the anterior skull at the junction of the frontal and ethmoidal bones [3]. The subgroup of nasofrontal encephaloceles herniates at the junction of the frontal and nasal bones resulting in deformation of the nasal bones and supraorbital ridge [2, 5, 12]. Here, we present a case of an open nasofrontal encephalocele requiring an immediate surgical reconstruction within the first 24 h of life.

## Case presentation

**Fig. 1:**
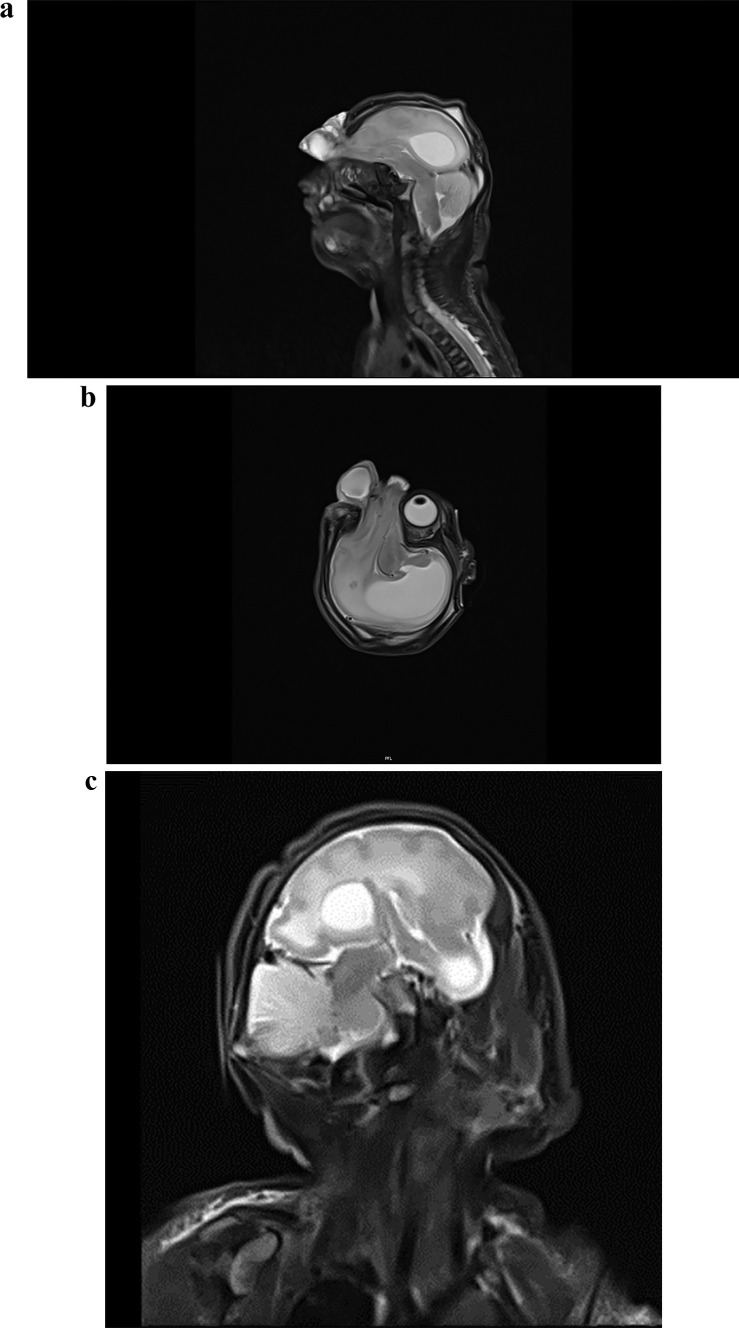
Preoperatively MRI Scans. (Sagittal, Axial and Coronal). In the T2-weighted image scans the encephalocele is identified together with the monoventricle. On the coronal slide the 90-degree hemispheric rotation is to be noted

**Fig. 2:**
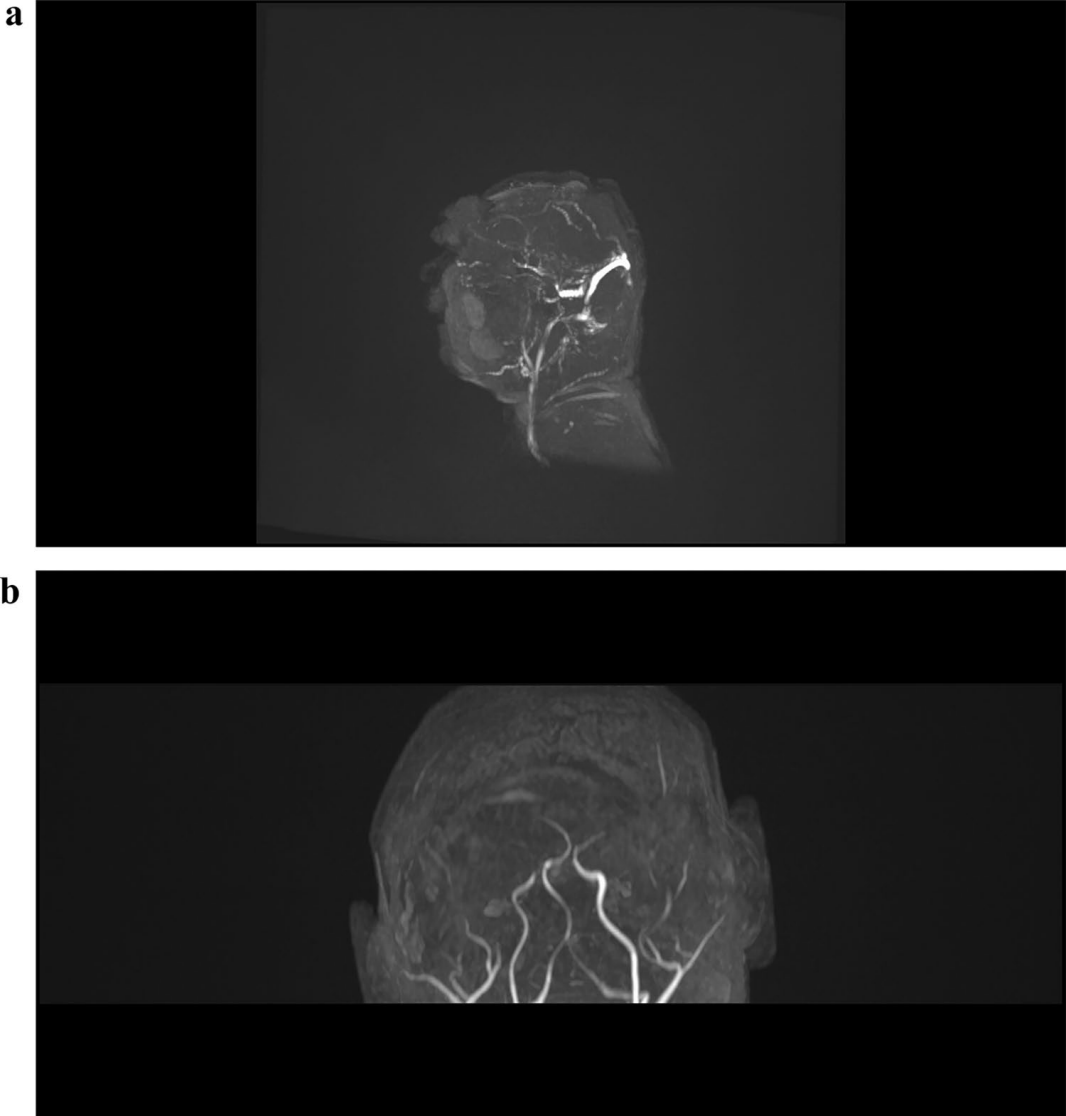
Preoperatively MR-Angiography

**Fig. 3:**
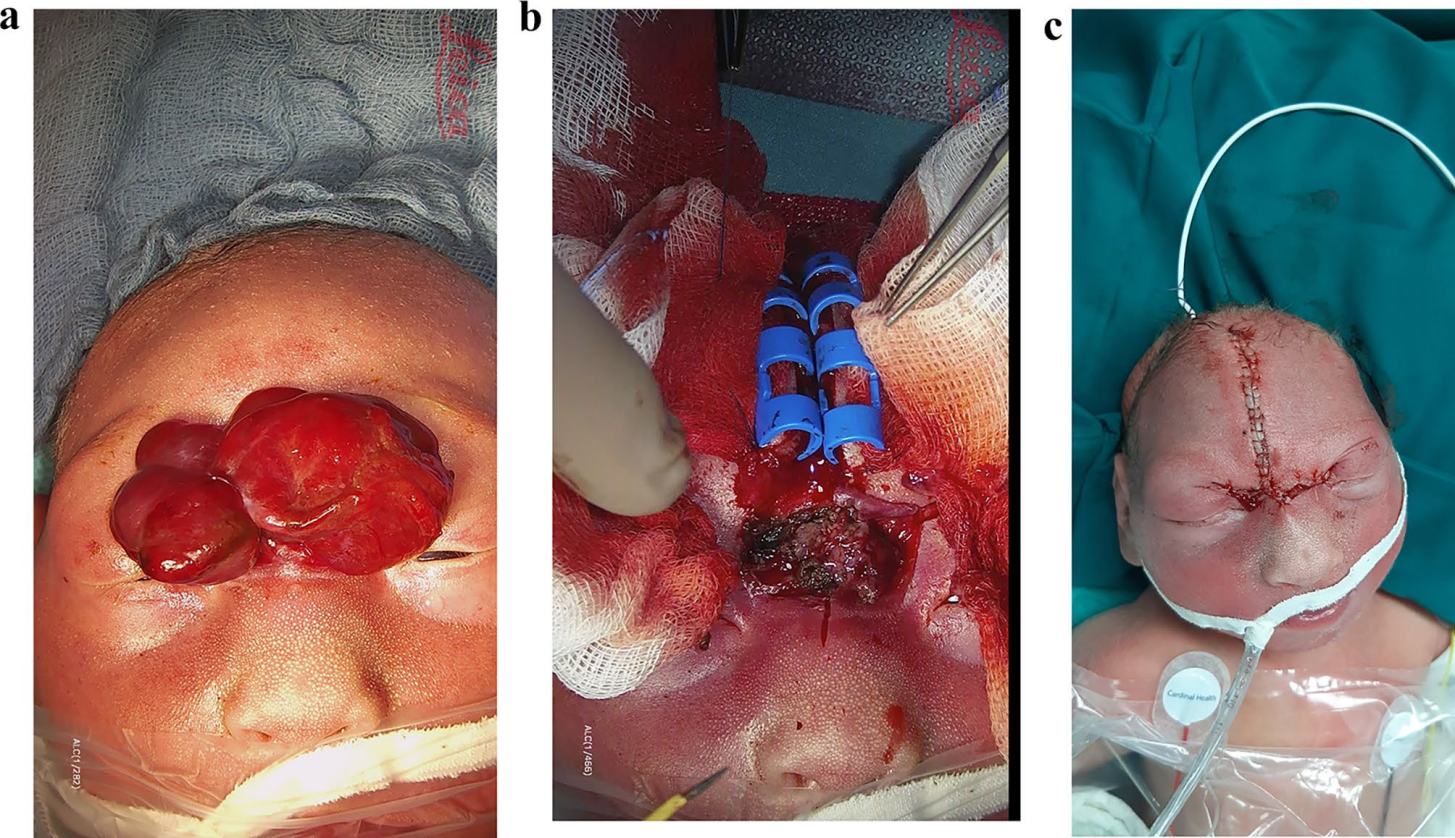
Intraoperative photographs showing the defect before the operation presenting the (**a**) nasofrontal encephalocele. Furthermore (**b**, **c**) presenting the midfacial incision and use of Raney Clips to avoid blood loss. The skin closure with 5-0 Monocryl sutures

**Fig. 4:**
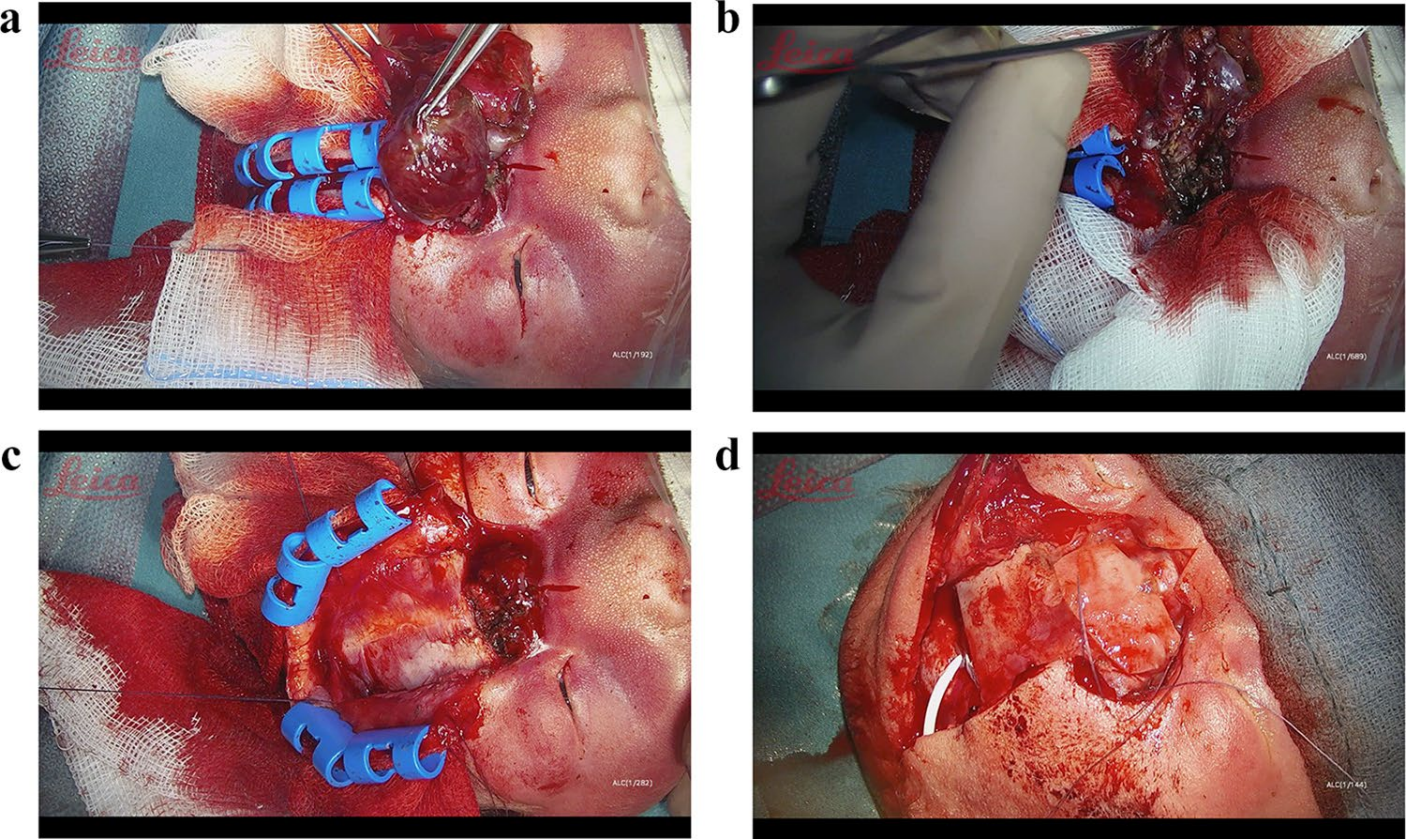
A series of images showing the intraoperative Steps. At first mobilising the Encephalocele, followed by resecting it en toto. The closure of the bony defect using a bone flap from the parietal bone. Lumbar Drainage used as an external ventricle drainage

**Fig. 5:**
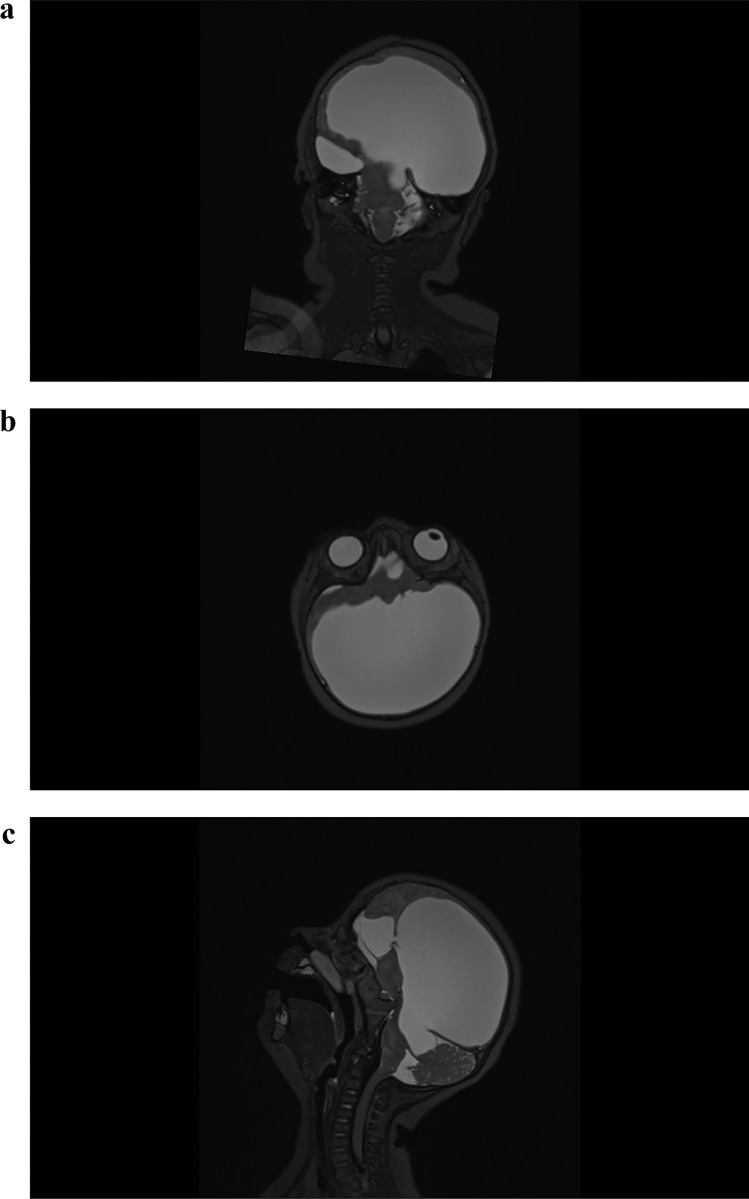
MRI Scan ( axial and coronal ) 6 months after Operation demonstrating the Hydrocephalus

Our male patient was born at 35 weeks + 6 days. During the fetal malformation scan, the family was informed about the encephalocele and decided to continue with the pregnancy. The delivery was performed by a caesarean section. The baby was immediately transferred to our University Hospital and the defect was secured in sterile covers. The patient was admitted in a stable, awake condition. The pupils were fully dilated and irresponsive to light. There was no apparent neurological deficit. We performed preoperatively an MRI and MR-angiography (Figs. 1 and 2), revealing an anatomical malposition of the hemispheres as well as an altered arterial supply. We decided for a mid-facial incision, reducing the bleeding and avoiding unnecessary injury in comparison to a bicoronal approach. The incision was made from the level of coronal suture and ending on nasion (Fig. 3). The entire operation was performed microsurgically. The herniated brain needed to be resected en bloc. The periorbital bone was exposed and explored. No bone defect was found (Fig. 4). Gradually, we gained access to the frontal basis where complete bony closure was confirmed. As expected, there was a broad dural defect. The closure was achieved using Galea periosteum. In order to reconstruct the bony defect, we harvested a 2-cm-wide autologous split calvarial bone graft, from the parietal bone on the left site. The bone graft was fixated over the frontal defect using fibrin glue. Strategically, we created a fontanel that was primarily non existing. Due to the uncertain status of CSF circulation and enlarged ventricles, there was a high risk for development of hydrocephalus. The fontanel gave access to the ventricular system. Furthermore, we inserted an external ventricular drainage (EVD) under ultrasound Guidance. The skin was closed using 5–0 Monocryl sutures.The patient was discharged 12 days after the operation in a stable clinical status. The postoperative course was uneventful. The EVD could be removed after 3 days in the absence of signs of hydrocephalus. The follow-up clinical examinations would be performed through pediatric neurosurgeons and pediatric neurologists. The patient developed therapy-resistent epileptic seizures that required treatment with a combination of five different anti epileptic drugs. An MRI-scan 6 months after surgery prevailed a growing hydrocephalus. Upon these clinical and radiological findings, the implantation of a ventriculoperitoneal shunt was performed (Fig. 5).

## Discussion

We present the second case of an open frontonasal encephalocele with pre and post MRI imaging. According to the pathogenesis, encephaloceles could be divided to primary and secondary (i.e., posttraumatic or iatrogenic origin). Although cases have been recorded since the sixteenth century, there is still a scarcity of knowledge on the exact causes and factors associated with the development of the disease. Many studies have determined these to be caused by a combination of genetic and environmental factors [13]. As mentioned, a primary open encephalocele presents a very rare entity, taking into consideration the availability of the extensive intrauterine examination of the fetus throughout pregnancy. The defect is usually located at the Foramen cecum, anterior to the cribriform plate of the ethmoid bone, and posterior to the frontal bone, within the frontoethmoidal suture. A primary understanding of the anatomy before surgery is essential. We achieved it with a series of MRI scans including MR angiography. As shown on the MRI scans, we identified a 90° anticlockwise rotation of the telencephalon. The unique anatomical variation was revealed including a singular ventricle, as well as the absence of transverse and sigmoid sinus. We were not able to identify the arteries of circle of Willis. The great responsibility of accompanying a neonate with an open encephalocele should be performed in clinical centers, where an experienced team of obstetricians, anaesthesiologists, neurosurgeons, and neonatologists work under the same roof. Surgery is prompted due to CSF leakage and exposed cerebral structures. The technical aspects of the operation, both from the anaesthesiological point of view and from our neurosurgical point of view, presented a high degree of challenge. The use of an EVD, and the creation of a fontanel (from removing a small bone from the parietal bone) gave us the great benefit of postoperatively controlling and excluding hydrocephalus. The ethical issue remains open to debate whether the operation was beneficial to our patient. From our point of view, the parents were aware of the situation of their unborn baby months since prenatal counselling and decided to carry on the pregnancy. During the informed consent, the parents were clear that a full treatment was desired, acknowledging the possible detrimental neurological development. Therefore, a conservative approach was not a treatment option.

## Conclusion

We present the second case of an open nasofrontal encephalocele, urging to a surgical intervention within 24 h after birth. Despite the early surgical intervention as well the uneventful postoperatively course, the detrimental neurological developmental delay could not be influenced.

## Data Availability

Data and material are available.

## Abbreviations

NTD: Neural tube defect
CSF: Cerebrospinal fluid
CNS: Central nervous system.
